# Description of the Content and Quality of Publicly Available Information on the Internet About Inhaled Volatile Anesthesia and Total Intravenous Anesthesia: Descriptive Study

**DOI:** 10.2196/47714

**Published:** 2023-11-02

**Authors:** Xinwen Hu, Bethany R Tellor Pennington, Michael S Avidan, Sachin Kheterpal, Nastassjia G deBourbon, Mary C Politi

**Affiliations:** 1 Department of Anesthesiology Washington University in St Louis School of Medicine St Louis, MO United States; 2 Department of Anesthesiology University of Michigan Ann Arbor, MI United States; 3 Magnolia Regional Health Center Corinth, MS United States; 4 Department of Surgery Washington University in St Louis School of Medicine St Louis, MO United States

**Keywords:** internet, general anesthesia, inhaled volatile anesthesia, total intravenous anesthesia, patient education, shared decision-making, surgery, information, decision-making, web-based, anesthesia, anesthesiology, anesthesiologist

## Abstract

**Background:**

More than 300 million patients undergo surgical procedures requiring anesthesia worldwide annually. There are 2 standard-of-care general anesthesia administration options: inhaled volatile anesthesia (INVA) and total intravenous anesthesia (TIVA). There is limited evidence comparing these methods and their impact on patient experiences and outcomes. Patients often seek this information from sources such as the internet. However, the majority of websites on anesthesia-related topics are not comprehensive, updated, and fully accurate. The quality and availability of web-based patient information about INVA and TIVA have not been sufficiently examined.

**Objective:**

This study aimed to (1) assess information on the internet about INVA and TIVA for availability, readability, accuracy, and quality and (2) identify high-quality websites that can be recommended to patients to assist in their anesthesia information-seeking and decision-making.

**Methods:**

Web-based searches were conducted using Google from April 2022 to November 2022. Websites were coded using a coding instrument developed based on the International Patient Decision Aids Standards criteria and adapted to be appropriate for assessing websites describing INVA and TIVA. Readability was calculated with the Flesch-Kincaid (F-K) grade level and the simple measure of Gobbledygook (SMOG) readability formula.

**Results:**

A total of 67 websites containing 201 individual web pages were included for coding and analysis. Most of the websites provided a basic definition of general anesthesia (unconsciousness, n=57, 85%; analgesia, n=47, 70%). Around half of the websites described common side effects of general anesthesia, while fewer described the rare but serious adverse events, such as intraoperative awareness (n=31, 46%), allergic reactions or anaphylaxis (n=29, 43%), and malignant hyperthermia (n=18, 27%). Of the 67 websites, the median F-K grade level was 11.3 (IQR 9.5-12.8) and the median SMOG score was 13.5 (IQR 12.2-14.4), both far above the American Medical Association (AMA) recommended reading level of sixth grade. A total of 51 (76%) websites distinguished INVA versus TIVA as general anesthesia options. A total of 12 of the 51 (24%) websites explicitly stated that there is a decision to be considered about receiving INVA versus TIVA for general anesthesia. Only 10 (20%) websites made any direct comparisons between INVA and TIVA, discussing their positive and negative features. A total of 12 (24%) websites addressed the concept of shared decision-making in planning anesthesia care, but none specifically asked patients to think about which features of INVA and TIVA matter the most to them.

**Conclusions:**

While the majority of websites described INVA and TIVA, few provided comparisons. There is a need for high-quality patient education and decision support about the choice of INVA versus TIVA to provide accurate and more comprehensive information in a format conducive to patient understanding.

## Introduction

More than 300 million patients undergo surgical procedures requiring anesthesia worldwide annually [1]. Inhaled volatile anesthesia (INVA) and total intravenous anesthesia (TIVA) are the 2 most commonly used standard-of-care general anesthesia administration methods. Insufficient evidence is available to establish which method is associated with superior patient experiences and outcomes. In the absence of robust comparative effectiveness trials evaluating patient experiences with each option, it is likely that most clinicians feel unable to discuss the differences from the patient’s perspective between these general anesthesia techniques, leaving patients who are interested in this comparison to seek information from the internet or other sources. For example, a recent survey noted that 40% of patients who have had surgery in the past 5 years were not included in the decision to choose INVA versus TIVA and almost half of these patients looked for information on their own about general anesthesia before their surgery. Of the 585 places searched, 412 (70%) were online websites [2]. Many patients report using the internet to learn more about their surgical procedures in general [3-5]. Enabling patients to be informed with the best available evidence is a critical component of high-quality patient care [6]. Information gathered from web-based sources can influence patients’ decision-making [7], so it is important to ensure patients are able to access accurate, comprehensible, and high-quality information.

Unfortunately, the majority of the websites on anesthesia-related topics are not comprehensive, updated, and fully accurate [8-15]. In addition, although some high-quality and accurate websites about anesthesia exist, they do not always rise to the top of the search engine results [14]. Moreover, most websites on anesthesia-related topics are written at a reading level above the American Medical Association (AMA) recommended level of sixth grade [8,10,11,15,16]. In fact, the median readability level is around a 13.5 (IQR 12.0-14.6) grade level, at which only about 62% of US adults can easily understand [11,17]. There are some available website quality certifications developed by national organizations or independent foundations, but these are not always indicative of content quality [10]. In addition, patients have expressed concerns with the process of searching for web-based information about general anesthesia. In a previous survey, 65% of patients who sought information about general anesthesia through web-based resources noted that it took a lot of effort to get the information they needed; 53% felt frustrated during their search [2].

Current studies have not sufficiently examined the quality and availability of web-based patient information about INVA and TIVA. The objective of this study was to assess publicly available information on the internet regarding both methods of general anesthesia administration for availability, readability, accuracy, and quality. We aimed to identify high-quality websites that can be recommended to patients to help assist them in making informed decisions about their anesthetic care.

## Methods

### Website Selection

Web-based searches were conducted using Google, the most commonly used search engine worldwide with a market share of over 90% [18-20]. All searches were performed in the United States from April 2022 to November 2022, using a browser with no stored cookies or browsing history to avoid generating personalized results. The following keywords were searched: “general anaesthesia,” “general anesthesia,” “anaesthesia,” “anesthesia,” “putting to sleep for surgery,” “propofol,” “intravenous anaesthesia,” “intravenous anesthesia,” “total intravenous anaesthesia,” “total intravenous anesthesia,” “inhaled volatile anaesthesia,” “inhaled volatile anesthesia,” “anaesthetic gases,” and “anesthetic gases.”

Each web page had to meet all of the following eligibility criteria to be included: (1) was displayed within the first 3 pages of search engine results when searching any of the keywords specified above, as over 90% of individuals do not look beyond the first 3 pages [19,20]; (2) was publicly available with no login required; (3) contained information on general anesthesia; (4) was intended for adult surgical patients; and (5) was written in English. Web pages were excluded if any of the following criteria existed: (1) they required logins, including subscriptions or free sign-ups; (2) they did not contain information about general anesthesia; (3) they were targeted toward medical professionals, defined as either websites that explicitly stated they were intended for use by medical professionals, or search results linked to books or scholarly journal papers that were not labeled as patient information pages; (4) they were written for pediatric patients and their parents; (5) they had a primary format of the video, social media, discussion board, question and answer forum, chat room, or personal blog; (6) they were identified by Google as a sponsored advertisement; or (7) they were written in any language besides English.

Each included web page and pages with the same domain name linked within 2 clicks were considered as a single website for subsequent coding and analysis. Linked web pages were excluded from the analysis if any of the exclusion criteria existed. External sites or references linked from eligible web pages were excluded. All embedded videos were excluded from the analysis.

### Ethical Considerations

As this study did not involve human subjects’ research, institutional review board oversight was not needed.

### Website Coding

A coding instrument (Multimedia Appendix 1 [21-41]) was developed based on the International Patient Decision Aids Standards (IPDAS) criteria for high-quality patient decision tools [21,22], adapted to be appropriate for measuring the quality of websites describing INVA and TIVA. Part I of the coding instrument was applied to all included websites that contained information on general anesthesia. This portion contained 28 items from the following categories: (1) basic definition and description of general anesthesia, (2) side effects and potential harms of general anesthesia, (3) what to expect with general anesthesia during the perioperative period, and (4) whether the website describes both inhaled and intravenous anesthesia as general anesthesia options. Items in part I included selected items from the IPDAS minimum standards of information necessary to support decisions and additional items adapted from previous research examples [19,20,42]. Part II of the coding instrument was adapted from the remaining IPDAS quality criteria needed to improve the quality of patient materials and was developed to evaluate the subset of websites that discussed both intravenous and inhaled anesthetic options. This section contained 29 items from the following areas: (1) comparison between INVA and TIVA, (2) qualitative or quantitative description of adverse event probabilities, (3) guidance for choosing between INVA and TIVA, and (4) evidence selection and disclosure. The coding instrument was discussed and iteratively revised by the study team to ensure clarity and agreement on definitions, coding approach, and items included. Once the team agreed on the items and coding process, the coder (XH) coded a sample website and clarified the remaining questions before coding the identified websites for analysis.

An item was checked if a website presented corresponding information in an accurate way, or if the criterium were satisfied per the rater’s judgment. All website coding was performed by a single researcher (XH) given the quantitative nature of the coding structure. Any ambiguity about coding was discussed among 3 of the authors (XH, BRTP, and MCP) and final decisions were made by consensus.

### Readability Assessment

The URLs of all web pages of each website were submitted to ReadablePro [43] for readability score calculation. Only the main body of the text was analyzed; header, footer, and references were excluded from the analysis. Readability for each web page was calculated with the Flesch-Kincaid (F-K) grade level [44] and the simple measure of Gobbledygook (SMOG) readability formula [45]. We randomly selected 10 web pages and manually calculated the F-K and SMOG readability to check accuracy; results from the ReadablePro calculation were consistent with manual calculations.

### Data Analysis

The frequency and percentage of websites that checked each item in the coding instrument were calculated. For each website, the number of items it checked in each category of part I and part II of the coding instrument were tabulated to determine the most comprehensive websites. For the readability of each website, the mean F-K grade level and mean SMOG score across all of its web pages were calculated. Descriptive statistics, including median, range, IQR, were then calculated for website mean F-K grade levels and website mean SMOG scores.

## Results

### Website Selection

**Figure 1:**
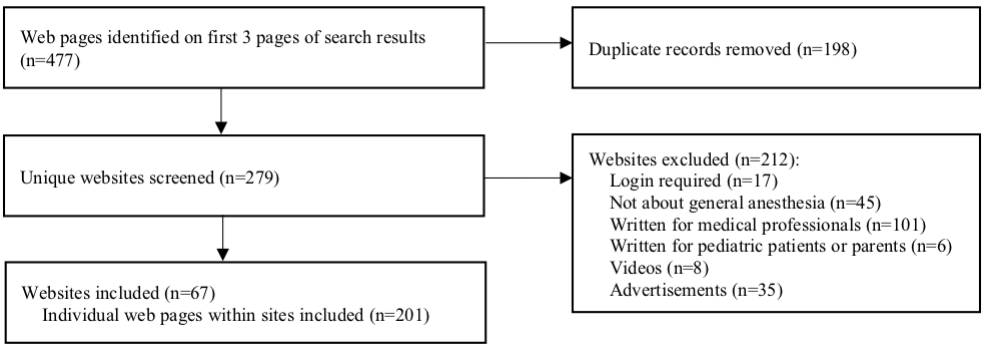
Inclusion or exclusion of websites identified in searches.

The website selection process is illustrated in Figure 1. A total of 477 web pages were identified on the first 3 pages of the search results. Of those, 198 duplicate records were removed. Of the remaining 279 websites, 212 were excluded because they required logins (n=17), did not contain information on general anesthesia (n=45), were targeted toward medical professionals (n=101), were written for pediatric patients and their parents (n=6), had a primary format of a video (n=8), or were identified by Google as advertisements (n=35). A total of 67 websites were formed from the included web pages and eligible pages linked within 2 clicks, with a total of 201 individual web pages included for coding and analysis.

### Website Coding

#### Content on General Anesthesia

##### Overall Description of General Anesthesia

All 67 websites were assessed using part I of the coding instrument (Table 1). Most of the websites provided a basic definition of general anesthesia (unconsciousness, n=57, 85%; analgesia, n=47, 70%) and described who administers general anesthesia (n=51, 76%). However, fewer than half of the websites discussed how general anesthesia is monitored during surgery (described monitoring of vital signs, n=33, 49%; described monitoring of “level of unconsciousness or awareness,” n=21, 31%; mentioned brain monitoring specifically, n=9, 13%).

Few websites discussed how general anesthesia works. Only 21 out of the 67 (31%) websites mentioned that general anesthesia provides control of the airway and breathing and allows for surgeries that affect breathing. A total of 17 (25%) websites mentioned muscle relaxation or immobility creates a controlled operative condition, 28 (42%) websites described the fact that general anesthesia works rapidly, and only 11 (16%) described the role of general anesthesia for surgeries that take a long time requiring longer sedation.

**Table 1:** Content of websites about general anesthesia (N=67).

Items				Number of websites, n (%)
**Basic definition and description of general anesthesia**				
	**Provided basic definition of general anesthesia**			
		Unconsciousness	57 (85)	
		Analgesia	47 (70)	
	Discussed who administers general anesthesia		51 (76)	
	**Discussed how general anesthesia is monitored during surgery**			
		Monitoring of vital signs	33 (49)	
		Assessment of “level of unconsciousness/awareness”	21 (31)	
		Brain monitoring (eg, processed electroencephalogram monitoring)	9 (13)	
	Provides control of airway and breathing or allows for surgeries that affect breathing		21 (31)	
	Provides muscle relaxation or immobility to prevent involuntary movements and create a controlled operative condition		17 (25)	
	Has a rapid onset of effect		28 (42)	
	Allows for surgeries that take a long time		11 (16)	
**Side effects and potential harms of general anesthesia**				
	**Common side effects**			
		PONV^a^	41 (61)	
		Chills or shivering	23 (34)	
		Sleepiness or confusion	34 (51)	
		Changes in heart rate and blood pressure	30 (45)	
	**Rare but serious adverse events**			
		Intraoperative awareness	31 (46)	
		Allergic reaction or anaphylaxis	29 (43)	
		Malignant hyperthermia	18 (27)	
		Propofol related infusion syndrome	10 (15)	
	**Risk factors for general anesthesia adverse events**			
		Risk factors for PONV	9 (13)	
		Risk factors for intraoperative awareness	17 (25)	
		Risk factors for malignant hyperthermia	9 (13)	
**What to expect before, during, and after surgery with general anesthesia**				
	**Before surgery, patient will meet with their anesthesia care team to…**			
		Review medical history	30 (45)	
		Discuss anesthesia options	23 (34)	
	Patient will need to fast before surgery		27 (40)	
	After anesthesia is administered, the patient will receive an endotracheal tube or alternative airway options		37 (55)	
	After the surgery is completed, anesthesia will be discontinued, and patient will regain consciousness		22 (33)	
	Some patients may take a longer time to wake up		2 (3)	
	Patient may have worse pain as the anesthesia wears off		9 (13)	
**Description of inhaled and intravenous anesthetics as general anesthesia options**				
	Described inhaled and intravenous anesthetics as general anesthesia options		51 (76)	

^a^PONV: postoperative nausea and vomiting.

##### Side Effects and Potential Harms of General Anesthesia

Around half of the websites described common side effects of general anesthesia such as postoperative nausea and vomiting (PONV; n=41, 61%), chills or shivering (n=23, 34%), sleepiness or confusion (n=34, 51%), and changes in heart rate and blood pressure (n=30, 45%). For rare but serious adverse events of general anesthesia, intraoperative awareness was described by 31 (46%) websites, allergic reactions or anaphylaxis by 29 (43%), malignant hyperthermia by 18 (27%), and propofol related infusion syndrome by 10 (15%). At least 1 risk factor for PONV was mentioned by 9 (13%) websites, 17 (25%) discussed risk factors for intraoperative awareness, and 9 (13%) discussed those for malignant hyperthermia.

##### Expectations for the Perioperative Period

Fewer than half of the websites described what to expect before surgery, including meeting with their anesthesia team to review medical history (n=30, 45%) and discuss anesthesia options (n=23, 34%) and fasting before surgery (n=27, 40%). The probable need for endotracheal intubation or alternative airway options was discussed by 37 (55%) websites. A total of 22 (33%) websites explicitly stated that anesthesia will be discontinued at the end of surgery for patients to regain consciousness, but only 2 (3%) mentioned the possibility of needing a longer time to regain consciousness. Only 9 (13%) websites helped set the expectation that patients may experience worsening pain as anesthesia wears off.

#### Content on INVA versus TIVA

##### Overview

Of the 67 websites analyzed, 51 (76%) distinguished inhaled versus intravenous anesthetics as general anesthesia options. These websites were further assessed with part II of the coding instrument (Table 2).

**Table 2:** Content of websites that described inhaled volatile anesthesia versus total intravenous anesthesia as general anesthetic options (N=51).

Items			Number of websites, n (%)
**Information**			
	Explicitly stated there is a decision that needs to be considered regarding using INVA^a^ or TIVA^b^ when general anesthesia is indicated		12 (24)
	**Mentioned the decision of using INVA vs TIVA depends on…**		
		Clinician’s preference	0 (0)
		Patient’s medical history	4 (8)
		Surgery or procedure requirements	1 (2)
		Patient’s preferences	0 (0)
	**Described positive features of INVA**		
		Standard of care for decades	8 (16)
		Predictable dose-response relationship	3 (6)
	**Described positive features of TIVA**		
		Standard of care for decades	7 (14)
		Lower risk of postoperative nausea and vomiting compared to inhaled agents	8 (16)
	**Described negative features of INVA**		
		Malignant hyperthermia	9 (18)
		Greenhouse gases or more atmospheric pollution compared to TIVA	6 (12)
	**Described negative features of TIVA**		
		Reactions to propofol, for example, allergic reaction, anaphylaxis, bacterial contamination leading to infection, and propofol infusion syndrome	9 (18)
	Compared the costs of INVA and TIVA		4 (8)
	Showed the negative and positive features of the 2 general anesthesia administration options with equal detail		12 (24)
	Made it possible to compare the positive and negative features of INVA versus TIVA		10 (20)
**Probabilities**			
	Provided numeric or qualitative descriptions of the probabilities of the adverse effects associated with INVA and TIVA		1 (2)
	Provided more than 1 way of viewing the probabilities		0 (0)
	Provided information about the levels of uncertainty around adverse event probabilities		1 (2)
**Values**			
	Explicitly stated shared decision making is an option for anesthesia		12 (24)
	Asked patients to think about what matters most to them		0 (0)
**Guidance**			
	Provided a step-by-step way to choose anesthesia method		0 (0)
	Included tools like worksheets or list of questions to use when discussing anesthesia options with a clinician		5 (10)
**Evidence**			
	Provided citations to the evidence selected		24 (47)
	Provided the date of publication or the date of last update		35 (69)
	Provided information about the update policy		3 (6)
**Disclosure**			
	Stated funding source and institutional affiliations		51 (100)
	Provided author or medical reviewer credentials		20 (39)

^a^INVA: inhaled volatile anesthesia.

^b^TIVA: total intravenous anesthesia.

##### Information Criteria

A total of 12 out of the 51 (24%) websites explicitly stated that there is a decision to be considered regarding whether to use INVA or TIVA when general anesthesia is indicated. A minority of these websites explained that such a decision depends on patients’ medical history (n=4, 8%) or surgery or procedure requirements (n=1, 2%), and no website mentioned that this decision may also factor in clinicians’ or patients’ preferences.

INVA was identified as standard of care by 8 (16%) websites and TIVA by 7 (14%). In terms of the pros and cons of INVA and TIVA, only 10 out of the 51 (20%) websites made any direct comparisons between the 2 general anesthesia options. A total of 8 (16%) websites mentioned TIVA is associated with a lower risk of PONV compared to INVA. A total of 3 (6%) websites explored the more predictable dose-response relationship of INVA. Few websites specifically associated malignant hyperthermia (n=9, 18%) or worse atmospheric pollution (n=6, 12%) with INVA, and only 9 (18%) mentioned any adverse drug reactions. A total of 4 (8%) websites provided information related to the costs of each method.

A total of 28 of the 51 (55%) websites did not provide any further information about INVA and TIVA beyond distinguishing between inhaled and intravenous anesthetics. A total of 11 (22%) websites provided some information about each option, but failed to do so in a balanced way with similar amounts of information for each. Only 12 (24%) websites discussed INVA and TIVA with enough detail to distinguish between them, and presented information about the 2 anesthetic options with an equal amount of detail.

##### Probabilities Criteria

Only 1 website provided a qualitative description of the probabilities of adverse effects associated with INVA or TIVA. It expressed the level of uncertainty around the adverse event probabilities, but failed to provide alternative ways of viewing the probabilities (such as graphs). None of the websites provided any quantitative description of adverse event probabilities associated with INVA or TIVA.

##### Values and Guidance Criteria

A total of 12 out of the 51 (24%) websites mentioned the concept of shared decision-making in planning anesthesia care, but none asked patients to think about which features of INVA and TIVA matter the most to them in the specific setting of choosing between the 2. No websites provided any step-by-step way to guide patients in choosing which general anesthesia administration method they prefer. Of the 51 websites, 5 (10%) websites provided a list of questions that patients can ask when discussing their anesthesia care with clinicians, but none of those questions was specifically developed to facilitate the discussion with clinicians about choosing between INVA and TIVA.

##### Evidence and Disclosure Criteria

A total of 24 (47%) websites provided citations to the evidence selected. A total of 35 (69%) provided the date of publication or the date of the last update, but only 3 (6%) provided information about their update policy to help patients assess whether the information is outdated. All websites (n=51, 100%) disclosed their institution affiliations and funding source and 20 (39%) provided the credentials of the authors or the medical reviewers.

### Readability Assessment

Website F-K grade level and SMOG score are summarized in Table 3. Of the 67 websites, the median F-K grade level was 11.3 (IQR 9.5-12.8; range 6.5-17.3), and the median SMOG score was 13.5 (IQR 12.2-14.4; range 10.3-19.0). All websites had readability levels above the AMA recommended level of sixth grade [16]. A considerable portion of websites (21% per F-K grade level and 57% per SMOG score) had readability levels ≥13, at which ≥38% of the adult population in the United States would have difficulty reading [17].

**Table 3:** Readability level of websites.

Readability	All websites (n=67)		Websites that distinguished inhaled from intravenous anesthesia (n=51)	
	Website F-K^a^ grade level	Website SMOG^b^ score	Website F-K grade level	Website SMOG score
Median readability level, median (IQR); (range)	11.3 (9.5-12.8); (6.5-17.3)	13.5 (12.2-14.4); (10.3-19.0)	11.2 (9.5-12.7); (6.5-15.6)	13.3 (12.0-14.3); (10.5-17.0)
Number (%) of websites with readability level ≥13, n (%)	14 (21)	38 (57)	11 (22)	28 (55)

^a^F-K: Flesch-Kincaid.

^b^SMOG: simple measure of Gobbledygook.

### Most Comprehensive Websites

Websites that checked the highest number of items in part I of the coding instrument about general anesthesia were Wikipedia.org [46,47], verywellhealth.com [48], healthline.com [49], and GoodRx.com [50] (Table 4). For these websites, 5-17 linked web pages were included per website. Websites that were less comprehensive but had ≤3 linked web pages were ClevelandClinic.org [51,52], MedicalNewsToday.com [53], NHS.uk [54,55], NHSinform.scot [56], and OUH.NHS.uk [57]. Regarding specific subcategories of information about general anesthesia, Wikipedia.org [46,47] and verywellhealth.com [48] covered the highest number of items in the category of a basic definition and description of general anesthesia. Websites that covered the highest number of items in the category of side effects and potential harms of general anesthesia were Wikipedia.org [46,47], GoodRx.com [50], ASAHQ.org [58], ClevelandClinic.org [51,52], Drugs.com [59,60], and verywellhealth.com [48]. In terms of the expectations for the perioperative period, websites that covered the highest number of items were verywellhealth.com [48], MayoClinic.org [61], NHS.uk [54,55], OUH.NHS.uk [57], and Patient.info [62]. Among the 51 websites that distinguished inhaled versus intravenous anesthetics, Wikipedia.org [46,47] and NYSORA.com [63] checked the highest number of information items in part II of the coding instrument about INVA versus TIVA.

**Table 4:** List of the most comprehensive websites.

Website name		Compared INVA^a^ versus TIVA^b^	Number of webpages	Mean F-K^c^ grade level	Mean SMOG^d^ score
**Most comprehensive websites about general anesthesia**					
	Wikipedia.org [46,47]	Yes	17	12.7	14.0
	Verywellhealth.com [48]	Yes	12	10.1	12.8
	Healthline.com [49]	Yes	5	9.5	12.3
	GoodRx.com [50]	Yes	9	9.3	11.9
**Less comprehensive websites about general anesthesia but easily browsed with fewer clicks**					
	ClevelandClinic.org [51,52]	Yes	3	10.5	13.0
	MedicalNewsToday.com [53]	Yes	3	9.6	12.0
	NHS.uk [54,55]	Yes	2	9.4	12.6
	NHSinform.scot [56]	Yes	1	9.1	12.5
	OUH.NHS.uk [57]	Yes	1	8.9	12.2
**Most comprehensive websites about a basic definition and description of general anesthesia**					
	Wikipedia.org [46,47]	Yes	17	12.7	14.0
	Verywellhealth.com [48]	Yes	12	10.1	12.8
**Most comprehensive websites about sides effects and potential harms of general anesthesia**					
	Wikipedia.org [46,47]	Yes	17	12.7	14.0
	GoodRx.com [50]	Yes	9	9.3	11.9
	ASAHQ.org [58]	Yes	13	11.3	14.1
	ClevelandClinic.org [51,52]	Yes	3	10.5	13.0
	Drugs.com [59]	Yes	20	9.2	10.9
	Verywellhealth.com [48]	Yes	12	10.1	12.8
**Most comprehensive websites about expectations for the perioperative period**					
	Verywellhealth.com [48]	Yes	12	10.1	12.8
	MayoClinic.org [61]	Yes	1	9.4	12.0
	NHS.uk [54,55]	Yes	2	9.4	12.6
	OUH.NHS.uk [57]	Yes	1	8.9	12.2
	Patient.info [62]	Yes	4	8.3	11.4
**Most comprehensive websites for the comparison between INVA and TIVA**					
	Wikipedia.org [46,47]	Yes	17	12.7	14.0
	NYSORA.com [63]	Yes	2	15.0	16.1

^a^INVA: inhaled volatile anesthesia.

^b^TIVA: total intravenous anesthesia.

^c^F-K: Flesch-Kincaid.

^d^SMOG: simple measure of Gobbledygook.

## Discussion

### Principal Results

Up to 80% of US adults have used the internet to search for health information [64], and many surgical patients have looked for information on general anesthesia on their own using web-based information [2]. Consistent with previous studies on websites on anesthesia-related topics [8-15], this study identified limitations in the availability of web-based information about general anesthesia. Although majority of the included websites provided a basic definition and description of general anesthesia, most failed to explain the benefits and drawbacks of general anesthesia compared with other potentially relevant choices (eg, sedation, regional anesthesia, and local anesthesia). More importantly, most websites failed to adequately inform patients of side effects associated with general anesthesia, especially the potential for rare but serious complications. Websites often failed to describe the process of preparing for and undergoing general anesthesia during the perioperative period. The lack of information in these areas compromises the ability of web-based resources to adequately inform patients about their anesthesia care.

In addition, websites were inadequate in aiding patients in making an informed decision about receiving INVA versus TIVA for general anesthesia. INVA and TIVA are both standard-of-care general anesthesia administration methods with insufficient evidence to establish the superiority of 1 over the other regarding patient experiences and outcomes. Given this uncertainty, the choice of INVA versus TIVA is well suited to shared decision-making [65,66]. Essential elements of shared decision-making include acknowledging that there is a decision to be made, discussing the risks and benefits of available options based on best-available evidence, and eliciting patient’s values and preferences [67-70]. A website useful for helping patients make an informed decision about receiving INVA versus TIVA should cover these elements. Although most websites distinguished inhaled versus intravenous anesthetics, fewer than half of them provided any further information and only a quarter explicitly stated that there is a decision to be considered regarding whether to use INVA or TIVA when general anesthesia is indicated. Although the relative advantages and disadvantages of INVA and TIVA require comparative effectiveness trials, some reliable evidence is available, which is summarized in part II of the coding instrument (Multimedia Appendix 1 [21-41]), and can be addressed by websites. However, the known comparative effectiveness evidence in relation to INVA and TIVA was presented by fewer than 20% of the websites. Only 24% of the websites described shared decision-making or incorporating patients’ preferences when planning anesthesia care, and no websites asked patients to think about what is important to them when choosing between INVA versus TIVA. No websites provided additional tools, resources, or links to facilitate this decision-making process.

Of note, although websites failed to provide comprehensive information about general anesthesia, the information that was included was highly accurate. No website presented any inaccurate information related to items in the coding instrument. However, over half of the websites failed to provide supporting evidence with citations and the credentials of their authors or medical reviewers.

All websites had readability levels above the AMA recommended level of sixth grade at which 99% of US adults can read [16,17]. Websites had a median F-K grade level of 11.3 (IQR 9.5-12.8) and a median SMOG score of 13.5 (IQR 12.2-14.4), consistent with the findings in a previous study about anesthesiology-related patient education materials on the internet [11]. About 90% of US adults attained an education level of high school graduate or higher, equivalent to a readability level of 12, while only 62% attained a grade level of 13 or above [17,71]. Therefore, a considerable portion of the US adult population may have difficulty reading many of the websites on general anesthesia.

Patients report that they are interested in visiting websites about anesthesia if recommended by their clinicians [3-5], so a goal of this study was to identify high-quality websites that can be recommended to patients to aid them in making informed decisions about their anesthetic care. Per the coding instrument used in this study, the websites [46-50] that provided the most comprehensive information about general anesthesia each had its information dispersed over many web pages, so that patients would need to click through linked pages extensively in order to gather all the information. All [48-50] except for Wikipedia.org [46,47] had readability levels below the median values of all websites analyzed. Despite being less comprehensive, ClevelandClinic.org [51,52], MedicalNewsToday.com [53], NHS.uk [54,55], NHSinform.scot [56], and OUH.NHS.uk [57] each condensed its information into ≤3 web pages, allowing for an easier browsing experience. In addition, their readability levels were comparable to, if not lower than, the most comprehensive websites. Overall, those websites that were less comprehensive but easier to browse through, especially the ones [53,54,56,57] that were easier to read, might be more suitable as supplemental patient education recourses that can be recommended to surgical patients.

In terms of websites that provided specific information comparing INVA and TIVA, Wikipedia.org [46,47] and NYSORA.com [63] were the most comprehensive. The former [46,47] had the problems aforementioned, while the latter [63] was highly technical and difficult to read. None of the websites provided comprehensive information with good readability and in a format that made it easy to directly compare INVA and TIVA, highlighting the need to develop patient education materials that address these deficits while summarizing the best evidence currently available. Moreover, consistent with past work [2], the limited availability of information on the internet about INVA and TIVA may reflect a pervasive view or culture that it is not necessary or important to involve patients in decisions surrounding anesthesia care, including the choice of INVA versus TIVA. It may also reflect the fact that there is limited evidence available regarding the comparison between INVA and TIVA, particularly in the setting of noncardiac surgeries, due to the lack of robust comparative effectiveness trials [23,24,72-83]. Future studies are needed to compare patient recovery experiences and outcomes after using INVA versus TIVA for general anesthesia. If a benefit were found to be associated with 1 method over the other, it would be relevant to patients, and should influence their desire to choose between the 2 general anesthesia administration methods.

### Limitations

This study had some limitations. First, although the search terms were designed to reflect both technical terminology and laypersons’ language, patients might search with keywords different than the ones used in this study, and obtain different search results. Second, each included web page linked within 2 clicks was considered as a single website for analysis in order to imitate the public’s general approach to web browsing. However, in some cases, as many as 20 web pages were included within a single website, whereas it is unlikely for patients to read as extensively to encounter all the information presented. Third, the website selection criteria were designed to capture all websites that patients are likely to encounter when searching for information on general anesthesia; it is possible that patients are aware of the potential for misinformation on the internet and focus instead on a selective subset of websites that they trust. Thus the percentage of higher-quality, more comprehensive websites may be underestimated compared to what patients actually read when they select a more restricted subset. However, the less stringent website selection criteria used in this study conferred a greater ability to identify areas in which information was lacking. Fourth, the keyword searches and the extraction of web page contents were conducted once for each keyword or web page at a single point in time. Website contents could be updated over time, which would not be captured by the cross-sectional approach used in this study. Fifth, all website coding was performed by a single author, so the interrater reliability of the coding instrument cannot be assessed. However, most items in the coding instrument were objective, and any ambiguity encountered during the coding process was discussed among the authors until a consensus was reached. Sixth, videos were excluded from the analysis to ensure the comparability among included websites, but they can be an important source of information requiring future studies to evaluate. Seventh, non-English websites were excluded from the analysis. Evaluating and improving websites and patient education materials written in other languages would be valuable for the large population of non–English-speaking patients. Finally, readability formulas have their limitations. Both formulas used in this study involve assessing the number of syllables [44,45]. The topic word “anesthesia” has 4 syllables and is considered “polysyllabic” per the definition of the SMOG formula [45]. The unavoidable use of such topic words in anesthesiology-related materials may bias the readability scores toward higher values without necessarily creating difficulty for patients to understand. Readability is not a perfect surrogate for comprehensibility, and future studies are needed to assess how well commonly used readability scores correlate with patient comprehension.

### Conclusions

Websites about general anesthesia can benefit from additional, more comprehensive information and text readability. While some websites on general anesthesia provided more comprehensive information compared to others, no website on the specific comparison between INVA and TIVA can aid patients in deciding with their clinicians about these anesthetic options. There is a need for high-quality patient education materials about general anesthesia, particularly on INVA versus TIVA, to provide comprehensive, accurate information in a format conducive to patient understanding.

## Appendix

Multimedia Appendix 1 Two-part coding instrument adapted from the International Patient Decision Aids Standards (IPDAS) minimal criteria.

## Abbreviations

AMA: American Medical Association
F-K: Flesch-Kincaid
INVA: inhaled volatile anesthesia
IPDAS: International Patient Decision Aids Standards
PONV: postoperative nausea and vomiting
SMOG: Simple Measure of Gobbledygook
TIVA: total intravenous anesthesia

## Data Availability

The data sets generated and analyzed during this study are available from the corresponding author upon reasonable request.
