# Supplementary material for: Description of the Content and Quality of Publicly Available Information on the Internet About Inhaled Volatile Anesthesia and Total Intravenous Anesthesia: Descriptive Study
Source: JMIR Perioper Med. 2023 Nov 2;6:e47714. doi: 10.2196/47714 (PMC10654911; doi:10.2196/47714)
Supplement: Multimedia Appendix 1 [file periop_v6i1e47714_app1.docx]

Table S1. 2-part coding instrument adapted from the International Patient Decision Aids Standards (IPDAS) minimal criteria [21,22].

| **Internet Information about General Anesthesia (INVA and TIVA)** | **Mentioned**  **Y/N** |
| --- | --- |
| **Part I. General Anesthesia** | |
| The website describes general anesthesia, when and how it is used, and how patients are monitored when receiving general anesthesia |  |
| - Basic definition or description of general anesthesia |  |
| - - Unconsciousness (e.g., “completely unconscious” or “putting patient in a sleep-like state”) |  |
| - - No sensation of pain |  |
| - Discusses who administers general anesthesia – anesthesiologist and/or certified registered nurse anesthetist (CRNA) and/or certified anesthesiologist assistant |  |
| - Discusses how general anesthesia is monitored during surgery |  |
| - - Monitoring of vital signs (e.g., heart rate, blood pressure, body temperature, and breathing) |  |
| - - Assessing whether patient stays unconscious (i.e. assessing “level of unconsciousness/awareness”) |  |
| - - Brain monitoring (e.g. processed electroencephalogram (EEG) monitoring) |  |
| The website discusses the benefits of general anesthesia overall. |  |
| - Provides control of airway and breathing / allows for surgeries that affect breathing |  |
| - Muscle relaxation/immobility prevents involuntary movements to create a controlled operative condition |  |
| - Rapid onset of effect |  |
| - Allows for surgeries that take a long time |  |
| The website discusses the risks/side effects of general anesthesia overall. |  |
| - Common side effects |  |
| - - Postoperative nausea and vomiting (PONV)[23] |  |
| - - Chills/shivering |  |
| - - Sleepiness/confusion |  |
| - - Changes in heart rate and blood pressure |  |
| - Rare but serious adverse events |  |
| - - Intraoperative awareness (being awake during surgery) |  |
| - - Allergic reaction/anaphylaxis to the drug used[25] |  |
| - - Malignant hyperthermia (rapid increase in body temperature, muscle contraction and muscle damage, and disturbances in metabolism)[26,27] |  |
| - - Propofol infusion syndrome (slow/stopped heart rate, with severe disturbances in metabolism, muscle damage, and/or enlarged liver)[28] |  |
| The website presents risk factors for general anesthesia complications. |  |
| - Risk factors for PONV (check “yes” if any of the following risk factors are mentioned)   - Female sex   - History of PONV or motion sickness   - Nonsmoking status   - Younger age   - Use of inhaled volatile anesthetic agents   - Postoperative opioid use   - Duration of anesthesia   - Type of surgery (cholecystectomy, gynecological surgery, laparoscopic surgery)[29] |  |
| - Risk factors for intraoperative awareness (check “yes” if any of the following risk factors are mentioned)   - Obesity   - History of intraoperative awareness   - Emergency surgery   - Type of surgery (obstetric, cardiac, thoracic)   - Use of neuromuscular blockade[24]   - Anesthesia depth monitoring by clinical signs only (i.e. neither end-tidal anesthetic gas nor processed EEG guided)[30]   - Conflicting evidence on female sex, younger age, ASA physical status[24,31] |  |
| - Risk factors for malignant hyperthermia (check “yes” if any of the following risk factors are mentioned)   - Patient or family has a genetic variant pathogenic for malignant hyperthermia susceptibility   - Personal or family history of malignant hyperthermia   - History of clinical myopathy or having a genetic variant of unknown significance involving genes implicated in malignant hyperthermia susceptibility (e.g., *RYR1, CACNA1S, STAC3)*   - History of otherwise unexplained, especially recurrent, rhabdomyolysis   - History of idiopathic hyperCKemia   - History of otherwise unexplained exertional heat illness[27] |  |
| The website describes what to expect before, during and after surgery with general anesthesia |  |
| - Before surgery, patient will meet with their anesthesia care team to |  |
| - - review medical history |  |
| - - discuss anesthesia options |  |
| - Patient will need to fast before surgery[32] |  |
| - After anesthesia is administered, the patient will receive a breathing tube (e.g., “insert a tube into the mouth and down the airway”) (or alternative airway options) |  |
| - After the surgery is complete, anesthesia will be discontinued, and patient will regain consciousness |  |
| - - Some patients may take a longer time to wake up |  |
| - Patient may have worse pain as the anesthesia wears off. |  |
| The website describes general anesthesia options - INVA vs. TIVA   - Defined as the use of inhaled gaseous agents vs. intravenous medications (or a combination of both) to achieve general anesthesia   *****Proceed to Part II only if this criterion is satisfied***** |  |
| **Part II. INVA vs. TIVA** | |
| **Information** | |
| The website explicitly states there is a decision that needs to be considered, i.e., using INVA (“gases”; including using inhaled agents either alone or in combination with IV medications) vs. TIVA (IV medications only) when general anesthesia is indicated. |  |
| - This decision depends on |  |
| - - clinician’s preference |  |
| - - patient’s medical history |  |
| - - surgery/procedure requirements |  |
| - - patient’s preferences |  |
| The website describes the positive features of INVA. |  |
| - Standard of care for decades |  |
| - Predictable dose-response relationship (predictable relationship between how much medication is given and how large the effect will be) |  |
| The website describes the positive features of TIVA. |  |
| - Standard of care for decades |  |
| - Lower risk of postoperative nausea and vomiting compared to inhaled agents[23] |  |
| The website describes the negative features of INVA. |  |
| - Malignant hyperthermia (rapid increase in body temperature, muscle contraction and muscle damage)[26,27] |  |
| - More atmospheric pollution compared to TIVA[33] |  |
| The website describes the negative features of TIVA.   - Reactions to propofol   - Or specifically mentions any of the following:   - allergic reaction/anaphylaxis[25]   - bacterial contamination of propofol resulting in infection[34,35]   - propofol infusion syndrome (slow/stopped heart rate, with severe disturbances in metabolism, muscle damage, and/or enlarged liver)[28] |  |
| The website compares the costs of INVA and TIVA. |  |
| The website shows the negative and positive features of the two general anesthesia administration options with equal detail (e.g., using similar fonts, sequence, presentation of statistical information). |  |
| The website makes it possible to compare the positive and negative features of INVA vs. TIVA by providing any of the following:   - side-by-side tables - statistical information using the same reference group - explicit verbal statements about which option has a higher/lower risk for a given feature |  |
| **Probabilities** | |
| If numeric probabilities are provided:  The website provides numeric probabilities of the adverse effects associated with INVA and TIVA.  If numeric information is not provided:  The website provides qualitative descriptions of the adverse event probabilities associated with INVA and TIVA (e.g., “rare” vs “common”, or “often” vs “less often”, or “frequent” vs “infrequent”, etc.).  *****If ‘No’ to both, skip the rest of the Probabilities section and mark as N/A***** |  |
| Adverse event probabilities are presented using the same time period for INVA and TIVA. |  |
| If numeric probabilities are provided:  Adverse event probabilities are presented using the same denominator for INVA and TIVA  If numeric information is not provided:  Not applicable/no numeric information about probabilities |  |
| The website provides more than 1 way of viewing the probabilities (e.g., words, numbers, graphs and other media formats).  (There is little evidence on which format is most effective in communicating risks. Some patients find it helpful to have multiple presentation formats while others perceive them as information overload.[36,37] Additive or alternative pictorial information may improve comprehension in people with low health literacy.[38]) |  |
| The website provides information about the levels of uncertainty around adverse event probabilities. For example,   - By giving a range, and/or - By using expressions such as   - “Our best estimate is …”   - “There is low certainty evidence on the effect of INVA vs. TIVA on postoperative delirium and postoperative cognitive dysfunction”[39]   - “Intraoperative awareness has an estimated overall incidence of about 1-2 in 1,000.[31,40,41] However, estimates vary and there has not been a high-quality randomized controlled trial that has compared the risk of intraoperative awareness associated with propofol TIVA vs INVA.”   - Etc. |  |
| **Values** | |
| Explicitly states shared decision making is a potential option in anesthesia care – e.g., “clinicians will discuss anesthesia options with you including the risks/benefits of each considering your health history and needs for the procedure.” |  |
| The website asks patients to think about which features of INVA vs TIVA matter most to them, implicitly (e.g., any mentioning of “patients’ preference”) or explicitly (e.g., asking patient to reflect on what matters the most to them). |  |
| **Guidance** | |
| The website provides a step-by-step way to choose which general anesthesia method the patient prefers. |  |
| The website includes tools like worksheets or list of questions to use when discussing anesthesia options with a clinician. |  |
| **Evidence** | |
| The website provides citations to the evidence selected. |  |
| The website provides the date of publication or the date of last update. |  |
| The website provides information about the update policy. |  |
| **Disclosure** | |
| The website provides information about the funding source and/or institution affiliations. |  |
| The website provides author/medical reviewer credentials. |  |
